# Frequency Invariability of (Pb,La)(Zr,Ti)O_3_ Antiferroelectric Thick-Film Micro-Cantilevers

**DOI:** 10.3390/s18051542

**Published:** 2018-05-13

**Authors:** Kun An, Xuechen Jin, Jiang Meng, Xiao Li, Yifeng Ren

**Affiliations:** 1 School of Electrical and Control Engineering, North University of China, Taiyuan 030051, China; s1715044@st.nuc.edu.cn (X.J.); lixiaoydx@nuc.edu.cn (X.L.); renyifeng@nuc.edu.cn (Y.R.); 2 Advanced Manufacturing Technology of Provincial Key Laboratory, North University of China, Taiyuan 030051, China; johnmeng@nuc.edu.cn

**Keywords:** antiferroelectric thick films, frequency shift, micro-cantilevers

## Abstract

Micro-electromechanical systems comprising antiferroelectric layers can offer both actuation and transduction to integrated technologies. Micro-cantilevers based on the (Pb_0.97_La_0.02_)(Zr_0.95_Ti_0.05_)O_3_ (PLZT) antiferroelectric thick film are fabricated by the micro-nano manufacturing process, to utilize the effect of phase transition induced strain and sharp phase switch of antiferroelectric materials. When micro-cantilevers made of antiferroelectric thick films were driven by sweep voltages, there were two resonant peaks corresponding to the natural frequency shift from 27.8 to 27.0 kHz, before and after phase transition. This is the compensation principle for the PLZT micro-cantilever to tune the natural frequency by the amplitude modulation of driving voltage, rather than of frequency modulation. Considering the natural frequency shift about 0.8 kHz and the frequency tuning ability about 156 Hz/V before the phase transition, this can compensate the frequency shift caused by increasing temperature by tuning only the amplitude of driving voltage, when the ultrasonic micro-transducer made of antiferroelectric thick films works for such a long period. Therefore, antiferroelectric thick films with hetero-structures incorporated into PLZT micro-cantilevers not only require a lower driving voltage (no more than 40 V) than rival bulk piezoelectric ceramics, but also exhibit better performance of frequency invariability, based on the amplitude modulation.

## 1. Introduction

Micro-actuators have evolved with the development for the high-precision manufacturing of modern micro/nano technologies with important requirements of high response speed, low driving voltage, and great output displacement [1]. Recently, it has been a priority of key technologies of ultra-precision machining to design and develop micro-actuators with new functional materials. Piezoelectric, magnetostrictive material, and shape memory alloys are three kinds of representative and hot research materials, because they demonstrate awareness and response, and can easily be intellectualized, integrated, and miniaturized [2,3]. Different functional materials in the structure and physical properties can give rise to broad applications for micro-actuators, and can solve certain difficult technical problems associated with integrated and intellectualized structures [4]. Therefore, a good functional material is expected to hold a promising prospect in the fields of micro-machining, biomedicine, robotics, aeronautics and astronautics [5,6,7], etc.

Antiferroelectric materials are among the most promising functional materials, because of their better switching characteristics and faster response speeds, with the response times as short as an order of 10^−7^ s [8,9,10]. AFE material shows superiority in terms of strain, compared with other functional materials [11,12]. Recently, research of AFE materials has focused on material of ceramic bulk, which requires a higher applying voltage for phase transition. A phase transition voltage of dozens of kilovolts per centimeter restricts the development of AFE ceramic bulk. In contrast, AFE thin films are broadly studied, including PbZrO_3_ and (Pb,La)(Zr,Sn,Ti)O_3_ [13,14,15]. At present, AFE thin film materials are rarely applied on a large scale for their limited dielectric properties and low electric-field-induced strains [16]. Considering that there is a meaningful change in the volume of antiferroelectric films during phase transition, the field-induced strain effect can arrive at 0.8% [17,18,19]. As a result, it is an urgent demand and a fundamental problem to integrate AFE materials into MEMS technology with the help of the giant field-induced strain and sharp phase switching characteristics. It will provide a wide application prospect to design and develop micro-actuators with fast response speeds, large response displacements, and good transduction performance [20].

At present, research on the field-induced strain of AFE materials is focused on both ceramic bulk and thin film. However, high driving voltage (i.e., of dozens of kilovolts per centimeter) leads to the limited application of AFE ceramic bulk during phase transition. On the other hand, the AFE thin film cannot meet the requirements of current micro-actuators for relatively poor dielectric properties, finite strain, and low compressive strength. It seems that the AFE thick film materials can be a key to solving the dilemma mentioned above, because of their high performance.

In our earlier work, the micro-cantilever structure based on the (Pb,La)(Zr,Ti)O_3_ (PLZT) thick film was fabricated successfully [21,22]. In this paper, the performance of the natural frequency shift of micro-cantilevers was studied, including vibration simulation and measurement, the relationship between driving voltage and natural frequency, and the transducing characteristics, which were tested and analyzed.

## 2. Testing Scheme and Performance Analysis

**Figure 1:**
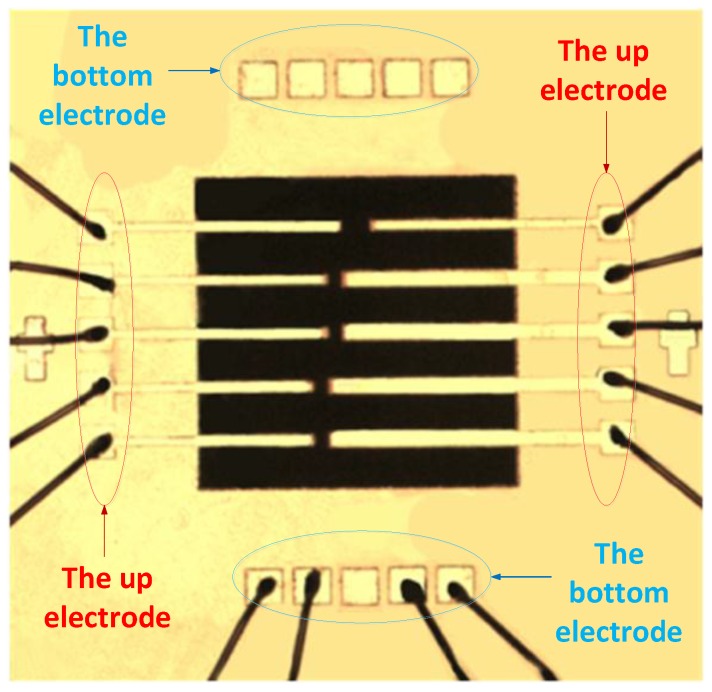
Wiring diagram of micro-cantilever.

**Figure 2:**
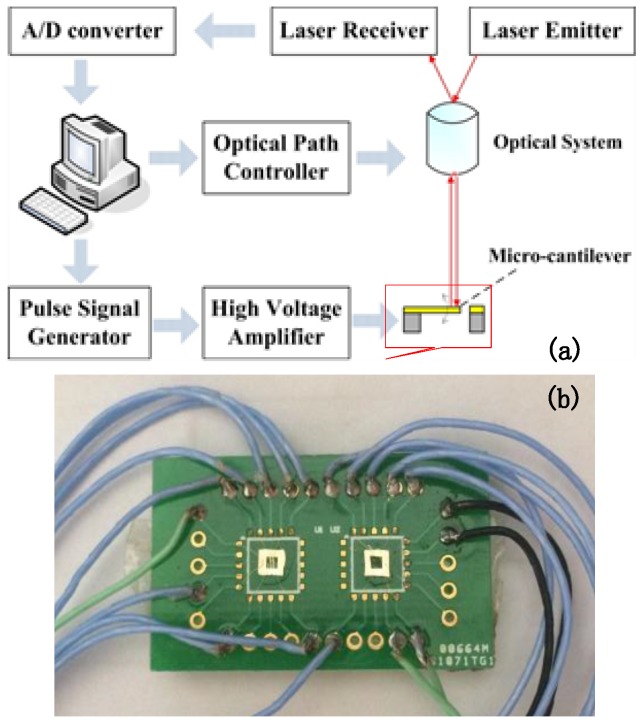
(**a**) measuring system diagram with the micro-system analyzer of MSA-400; (**b**) the encapsulation shell in the Printed Circuit Board.

Large area PLZT AFE thick films were fabricated on silicon substrates by heterogeneous integrated manufacturing [23]. The wiring diagram of micro-cantilever is shown in Figure 1. The micro-system analyzer (MSV-400, POLYTEC, Waldbronn, Germany) shown in Figure 2a was adopted to test the vibration behavior of PLZT micro-cantilevers with a high-voltage amplifier (HA-400, PINTEK, New Taipei City, Taiwan). There were 10 gold-welded PLZT micro-cantilevers with conducting wires, and the other end of wires in Figure 2b were welded to the special encapsulation shell in the Printed Circuit Board.

**Figure 3:**
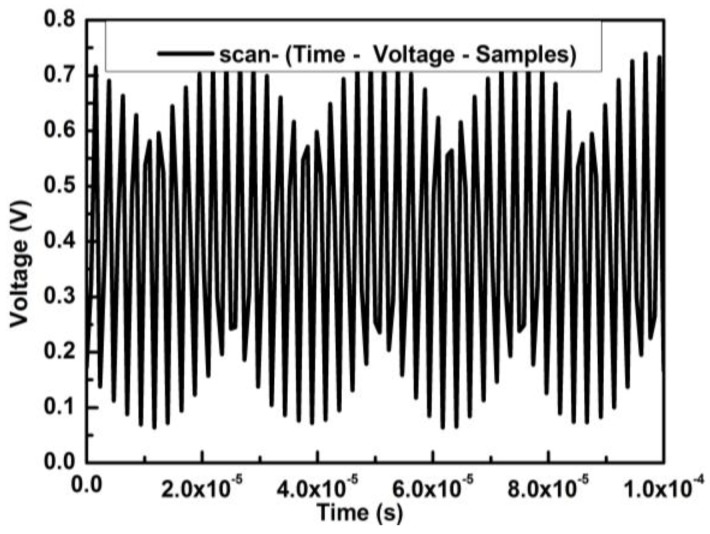
Original sweep signal of periodic chirp.

The linear frequency-modulated signal of MSA-400 was used for excitation with 1 V of both original amplitude voltage and offset voltage; thus, the sweep signal curve was plotted with time varying from 0 to 10^−4^ s in Figure 3.

The voltage excitation signals loaded onto the cantilever with the voltage amplifier were 10 V, 15 V, 20 V, 30 V and 35 V, respectively. The sampling frequency of *f*_s_ = 200 kHz was set to satisfy the range of vibration resonance frequency of the micro-cantilever, and the maximal sampling number of *N*_s_ = 6400 was to guarantee the minimal frequency resolution Δ*f*_s_,

(1) $$\Delta f_{s}=\frac{f_{s}}{N_{s}}=\frac{200\;\mathrm{kHz}}{6400}=31.25\;\mathrm{kHz}$$

**Figure 4:**
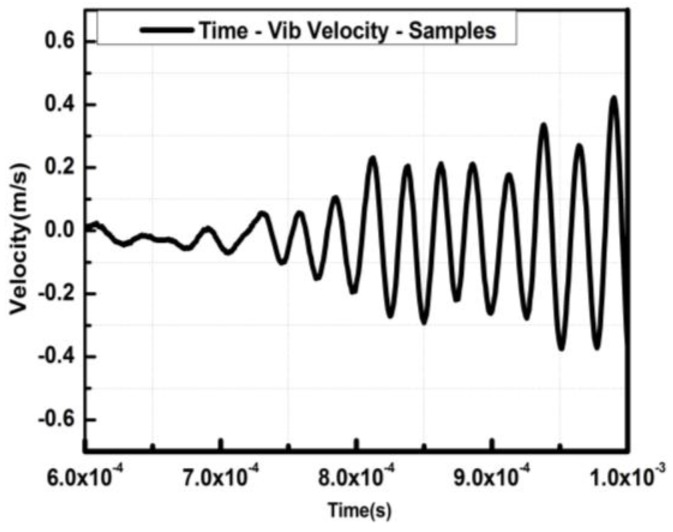
*V*elocity-*T*ime curve under 20 V chirp.

**Figure 5:**
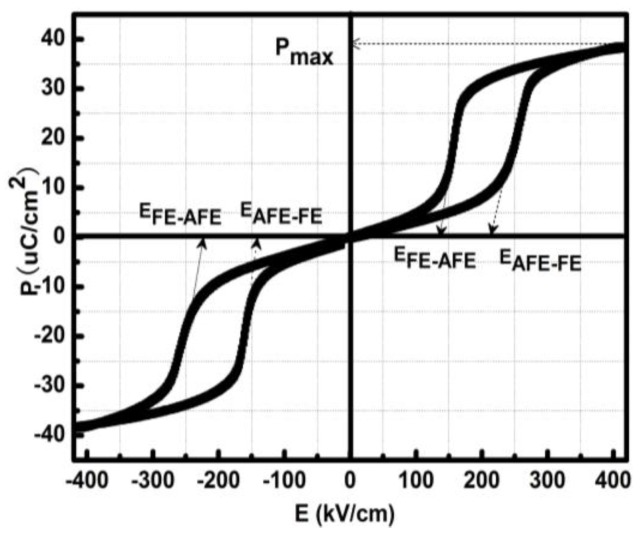
*P*olarization-*E*lectric (*P-E*) field density curves of antiferroelectric thick film.

Figure 4 shows a *V*elocity-*T*ime curve under the effective driving voltage signal of 20 V, where the single measuring point on the tip of micro-cantilever with 500 μm (L) × 40 μm (W) × 10 μm (H) was selected as the reference. The cantilever vibration under the chirp excitation is equivalent to the forced harmonic one, and thus, the resonant frequency equals the forced frequency. In this circumstance, the vibration period *T* is,

*T* = 1/*f* (2)

where *f* is the resonant frequency and the forced vibration velocity *v* = *2πfs*, and *s* is the displacement amplitude. The harmonic relationship among the vibration velocity, displacement amplitude and sweep frequency was verified, as shown in Figure 4 and Figure 5.

Figure 5 provided a typical double hysteresis loop square-like of PLZT thick films with a testing frequency of 100 kHz, where the saturated polarization of *P_s_* = 40 μC/cm^2^ corresponded to the electric field intensity of the AFE-FE phase transition of *E_F_* ≈ 200 kV/cm at a temperature of 60 °C. Thus, the theoretical driving voltage *U* was

*U* = *E_F_ × d* ≈ 200 kV/cm × 2 μm = 40 V (3)

where *d* was the PLZT-film thickness of the micro-cantilever.

## 3. Results and Discussions

**Figure 6:**
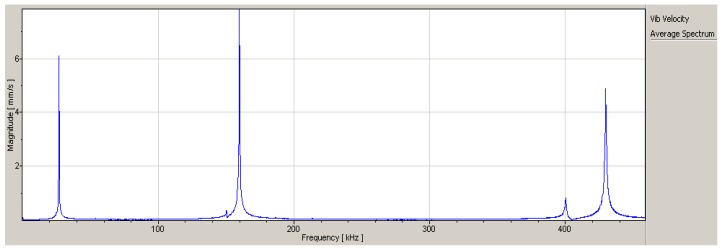
Velocity-Frequency curves under the sweeping mode.

Figure 6 presents the relationship between frequency and velocity of the micro-cantilever. The resonance frequencies of the first three orders were 26875 Hz, 159844 Hz and 430156 Hz.

**Figure 7:**
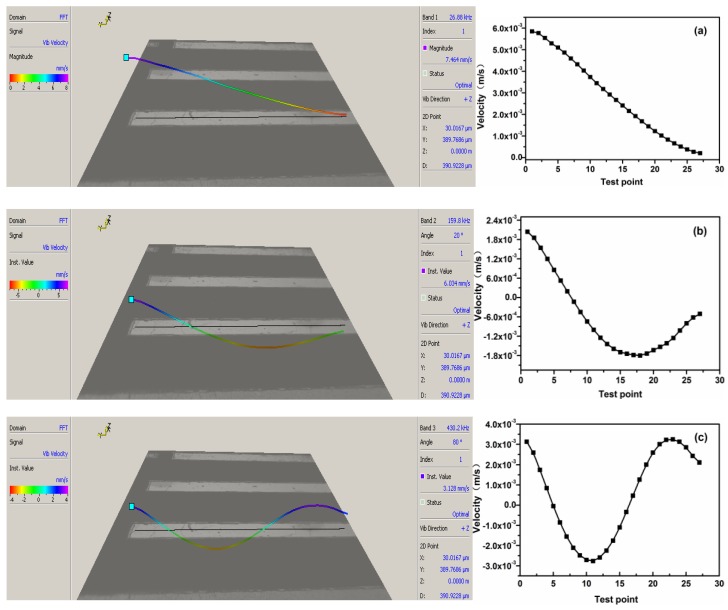
(**a**) the 1st order, (**b**) the 2nd order and (**c**) the 3rd order of vibration simulation (left) and measurement (right) of the micro-cantilevers.

Firstly, there was a small peak near 400 kHz, but it was not usually regarded as the resonance frequency, because its velocity was far less than other resonance velocities. Secondly, Figure 7a–c displayed the velocity curves of resonance characteristics with 25 test points of the micro-cantilever at the first three order frequencies. The vibration velocity at the 2nd-order frequency was larger than that at the 1st order, yet the integrated displacement was smaller. In contrast, the vibration velocity and displacement at the 3rd-order frequency was contrary to those of the 2nd order.

**Figure 8:**
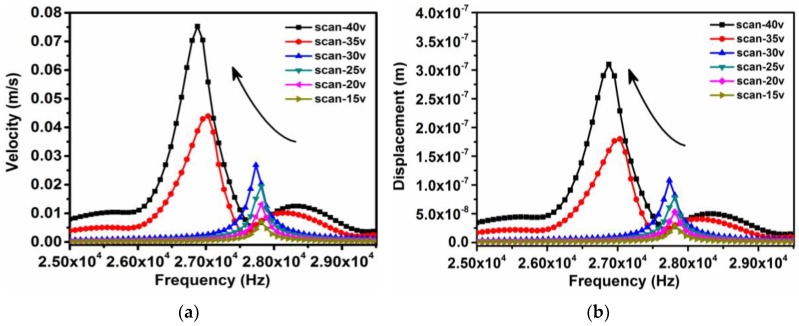
Resonance curves of micro-cantilever tip under different sweep voltages. (**a**) Displacement resonance curve, (**b**) Speed resonance curve.

**Figure 9:**
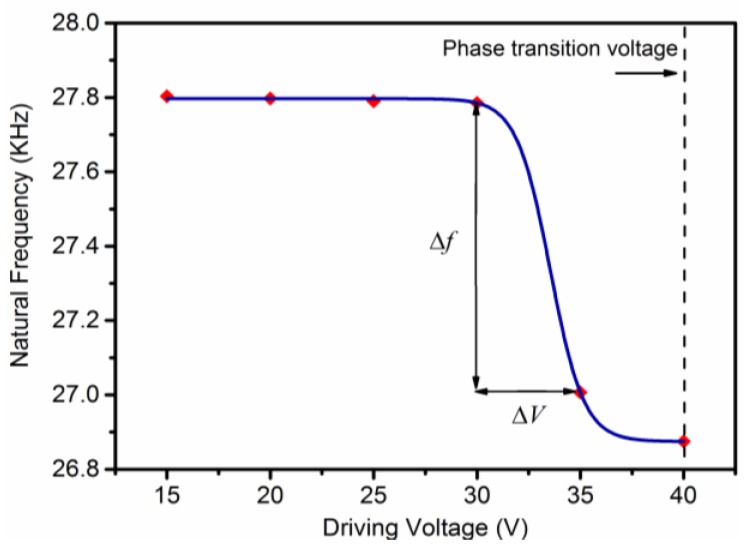
Velocity-Frequency curves under the sweeping mode.

**Figure 10:**
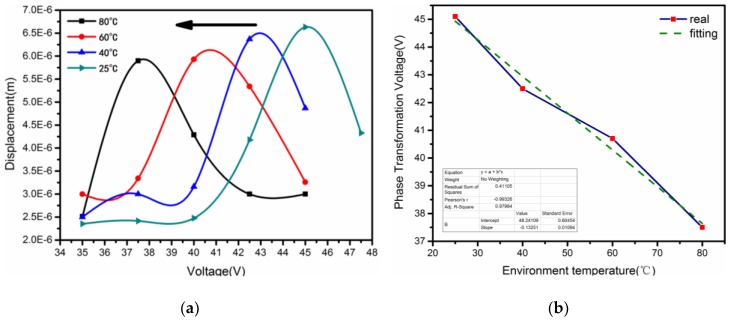
Relationship among displacement, phase transition voltage and temperature. (**a**) *Displacement-Voltage* curves, (**b**) *Phase Transformation Voltage-Environment temperature* curves.

The micro-cantilever vibration results in frequency domain were plotted in Figure 8, when loading different amplitudes of excitation voltages, such as 15, 20, …, 40 V. There were different resonance peaks when sweeping the frequency from 0 to 100 kHz. As a result, two resonant intervals were clearly seen, and are shown in Figure 9. One interval was the steady resonant frequency of *f*_1_ *≈* 27.80 kHz under a driving voltage of less than 30 V, while the other was located in a small range of *f*_2_ ≈ 26.88~27.01 kHz when applying the driving voltage from 35 to 40 V. Figure 10 showed the relationship between the driving voltage and natural frequency, where the maximal frequency shift Δ*f* and corresponding differential voltage Δ*V* before the phase transition could be read from the figure, Δ*f* = 0.778 kHz and Δ*V* = 5V. Thus, the rate *τ* of frequency change to voltage change can defined as the factor by which frequency tuning ability may be measures:

(4) $$\tau =\frac{df}{dV}\approx \frac{\Delta f}{\Delta V}=155.6\;\mathrm{Hz}/V$$

The frequency decrement is about 156 Hz for an increase of 1 V applied in the PLZT micro-cantilever when it is within the sensitive range from 30 to 35 V.

It is known that natural frequency is an intrinsic property for invariable structures made of given materials, regardless of any change in external conditions. A large increment of volume exists when the phase of micro-cantilever transforms from AFE to FE, which causes a measurable frequency decrement in macroscopic view (Figure 9), and a relative stress of phase transition between the AFE layer and the silicon layer in microscopic view. This phenomenon can be verified by examining the resonant response characteristics of the phase-transition-induced strain under the periodic excitation voltage.

In the electric-thermal coupling field, the maximal sweep displacement can indicate whether the micro-cantilever reaches phase transition. When the temperature varies in 25 °C, 40 °C, 60 °C and 80 °C, the driving voltage corresponding the maximal resonant displacement was 45.1 V, 42.5 V, 40.7 V and 37.5 V, respectively. There was a small voltage decrease (−7.6 V) of phase transition with the different temperature in Figure 10a, and the voltages of phase transition showed good linear variation (R^2^ = 0.9866), shown in Figure 10b. This linearity implied that the electric-thermal coupling field could cause roughly an equal effect when the PLZT micro-cantilever reached the phase transition, which was also verified in our earlier work [23].

Let us assume a general case that a PLZT ultrasonic transducer works before phase transition from AFE to FE at room temperature. When working for a long period of time, a temperature increase occurs around the transducer, even if it is equipped with a cooling system. The transducer may work ineffectively, owing to the decrease of natural frequency caused by the increasing temperature. Under these circumstances, the varied natural frequency has no need to be tracked by adjusting the frequency of driving voltage, but rather, tunes the voltage amplitude within a small range, as shown in Figure 9, in order to recover the original frequency. That is to say, the decreasing strain induced by the electric field can be treated as the compensation for the increasing strain induced by the temperature field. It is such a useful characteristic for ultrasonic transducers, if we are to avoid using expensive hardware, to generate variable frequency signals.

## 4. Conclusions

Based on the effect of phase-transition-induced strain, the PLZT micro-cantilever can be driven by chirp signals only from 15 V to 40 V under a steady temperature field, which is far less than those of piezoelectric ceramics which require hundreds of driving voltages. Thus, a natural frequency shift exists at about 0.8 kHz, and a frequency tuning ability at about 156 Hz/V, before phase transition. Since the characteristic of electric-based frequency shifts can compensate for the decreasing natural frequency of the PLZT micro-cantilever under the increasing temperature field, it can solve the failure problem of supersonic transducers made of PLZT thick films by simply tuning the amplitude, instead of the frequency, of the driving voltage. The characteristics of AFE thick films mentioned above can promote the development of micro-nano actuators based on both the low driving voltage, and good excellent performance of frequency invariability, only through a method of amplitude modulation.
